# COVID-19 e Eventos Coronários Agudos – Danos Colaterais. Um Relato de Caso

**DOI:** 10.36660/abc.20200329

**Published:** 2020-06-29

**Authors:** Luiz Eduardo Fonteles Ritt, Mateus S. Viana, Gustavo Freitas Feitosa, Adriano Martins de Oliveira, Fabio Solano Souza, Eduardo Sahade Darzé

**Affiliations:** 1 Hospital Cardio Pulmonar SalvadorBA Brasil Hospital Cardio Pulmonar,Salvador, BA - Brasil; 2 Escola Bahiana de Medicina e Saúde Pública SalvadorBA Brasil Escola Bahiana de Medicina e Saúde Pública,Salvador, BA - Brasil

**Keywords:** Infarto do Miocárdio com Supradesnível de ST, Coronavirus, Pandemia, Pânico, Mêdo, Cineangiografia, Ecocardiografia/métodos, Fatores de Risco

*“O medo que tens, Sancho, faz com que nem vejas nem ouças direito — disse dom Quixote —, porque um dos efeitos do medo é perturbar os sentidos e fazer com que as coisas não pareçam o que são.”*

Miguel de Cervantes em Dom Quixote

Paciente do sexo masculino, 49 anos, hipertenso há 8 anos, dislipidêmico, com histórico familiar de doença arterial coronariana (pai cursou com infarto aos 60 anos), vinha em uso de olmesartana 40 mg e rosuvastatina 10 mg ao dia até 10 dias antes da admissão hospitalar, quando suspendeu a olmesartana pelo receio de que esta droga facilitasse a infecção por SARS-CoV-2.

Na manhã de 02/04/2020 cursou com desconforto torácico retroesternal intenso e sensação de dispneia, desencadeados ao esforço menor e que cessaram com o repouso e recorreram em menor intensidade ao longo do dia. Preocupado com a possibilidade de infecção por SARS-CoV-2, isolou-se, monitorou curva térmica e usou paracetamol por conta própria. Não registrou febre. No dia seguinte, houve recorrência da dor torácica, agora com irradiação para ombros, associada à sudorese e dispneia. Devido à sudorese, ficou ainda mais preocupado com a possibilidade de SARS-CoV-2 e telefonou para um infectologista que o orientou, caso os sintomas persistissem ou recorressem, a procurar um atendimento de emergência. Ao longo do dia permaneceu isolado e realizando curva térmica. Referiu que “apenas a possibilidade de Coronavírus passava pela cabeça”.

Na manhã de 04/04 cursou com piora da dor, sudorese mais intensa e resolveu procurar emergência. Foi triado como possibilidade de síndrome coronariana aguda (SCA), mas não aceitou fazer os exames por não querer ficar no setor onde haviam outros pacientes, saindo à revelia. No caminho de volta à residência, os sintomas intensificaram, sudorese mais profusa e dispneia, mudando o curso do domicílio para nosso hospital, onde apresentou-se com taquicardia sinusal (FC 108 bpm), PA sistólica de 176 mmHg, saturação de O2 98% e temperatura de 36,4oC. Eletrocardiograma revelou supradesnível de segmento ST em V5, V6, D1, AVL ( Figura 1 ), configurando infarto agudo do miocárdio com supradesnível de ST (IAMcSST). O paciente foi submetido à cineangiocoronariografia e angioplastia primária de artéria descendente anterior em terço médio com um tempo porta-balão de 57 minutos ( Figura 2 ). Ecocardiograma mostrou disfunção sistólica de grau leve, devido à acinesia de toda a região apical e do segmento médio da parede anterior, com fração de ejeção de 45% através do Método de Simpson. Pico de troponina I de alta sensibilidade foi de 21.424 ng/L. O paciente evoluiu sem complicações e obteve alta após 3 dias de internação hospitalar. A Figura 3 mostra a sequência temporal dos fatos até o diagnóstico do IAMcSST.

**Figura 1:**
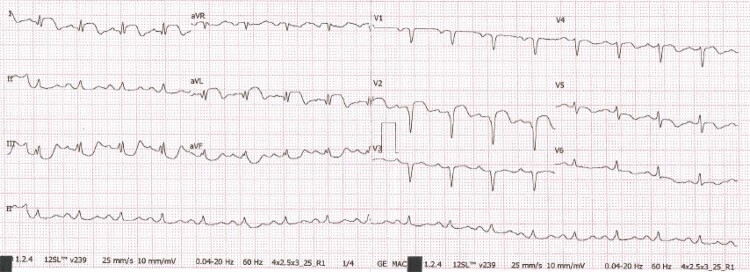
– *Eletrocardiograma da admissão*

**Figura 2:**
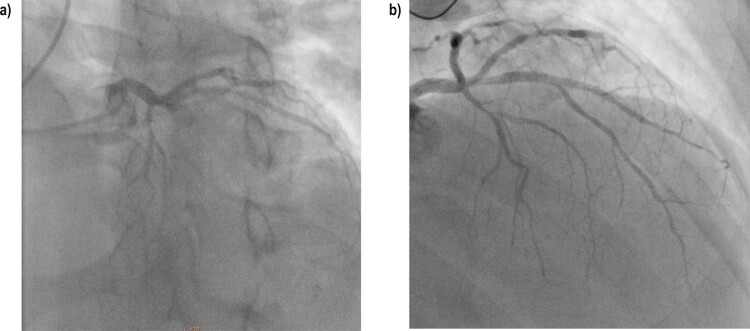
– *Cineangiocoronariografia representando: a) artéria coronária descendente anterior ocluída e b) após angioplastia primária.*

**Figura 3:**
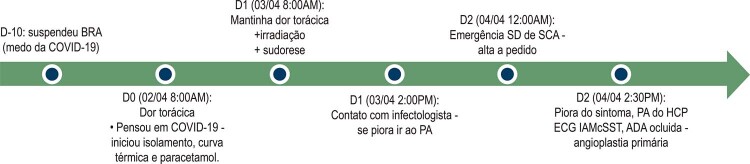
– *Linha do tempo desde o início dos sintomas até o diagnóstico do infarto do miocárdio. BRA, bloqueador renina angiotensina; PA, pronto atendimento; SD, suspeita diagnóstica; SCA, síndrome coronariana aguda; HCP, Hospital Cárdio Pulmonar; ECG, eletrocardiograma; IAMcSST, infarto agudo do miocárdio com supradesnível de segmento ST; ADA, artéria descendente anterior.*

## Discussão

Com a pandemia de SARS-CoV-2, períodos de quarentena têm sido declarados em diversas cidades do Brasil e do mundo, e indivíduos têm sido orientados a manter o distanciamento social para conter a rápida disseminação do vírus. O medo extremado de adquirir a infecção pode fazer com que sintomas típicos de uma SCA sejam negligenciados ou atribuídos erroneamente a outras causas menos prováveis, retardando seu tratamento e impondo riscos evitáveis à vida dos pacientes. Relatamos um caso típico de SCA em um paciente com fatores de risco para doença aterosclerótica, que, dirigido pelo pânico da COVID-19, foi incapaz de reconhecer a natureza dos sintomas, adiando sua ida à emergência até o momento em que a dor torácica se tornou intolerável. Adicionalmente, também por receio da infecção pelo SARS-CoV-2, o paciente suspendeu o uso do bloqueador do receptor da angiotensina (BRA). Apesar de um tempo porta-balão de 57 min, em decorrência do prolongado tempo de isquemia, o paciente evoluiu com disfunção sistólica do ventrículo esquerdo, ainda que assintomática.

### Atraso no reconhecimento e atendimento do infarto agudo do miocárdio

O infarto agudo do miocárdio (IAM) é a emergência médica que mais mata no mundo, possui uma incidência de 43 – 144 por 100.000 pessoas/ano e uma mortalidade hospitalar de 4 - 12%.^1^ A angioplastia primária, especialmente quando instituído nas primeiras 12 horas de início dos sintomas, é considerado o padrão-ouro de tratamento.^1 , 2^ O tempo porta-balão é indicador de qualidade de tratamento no contexto do IAM. Não menos importante é um curto tempo entre o início dos sintomas e chegada ao hospital. Diferente dos tempos de atendimento após a chegada no hospital que podem ser otimizados por fluxos e protocolos internos, o tempo até a chegada ao hospital é quase exclusivamente dependente da percepção do paciente e valorização de suas queixas.

A pandemia de SARS-CoV-2 trouxe outras perspectivas de abordagem desta patologia, considerando o potencial risco de contaminação em ambiente de hemodinâmica, cujos procedimentos podem necessitar de uma maior invasividade, com uma inadequação do ambiente para o controle do espalhamento viral e segurança dos profissionais de saúde.^3^ Publicação recente do epicentro desta pandemia pondera, inclusive, a possibilidade de terapia trombolítica para os casos confirmados com sintomas respiratórios da doença.^4^ .

O caso em questão ilustra um outro cenário da pandemia de SARS-CoV-2 tão preocupante quanto a própria pandemia per se. Estudos previamente publicados em outras epidemias virais sugerem um aumento da ocorrência de infarto do miocárdio, com maior propensão à inflamação e instabilidade de placa ^5^ e este também aparenta ser o racional para a infecção por SARS-CoV-2.^6^ Entretanto, relatos em diferentes centros mundiais apontam para uma redução na frequência de admissões por infarto, com estudo observacional apontando para uma queda de 40% no atendimento do infarto com supradesnível do segmento ST, com um discreto aumento na taxa de trombólise.^7^ Esta queda paradoxal pode estar associada a uma redução da procura destes pacientes a unidades de pronto-atendimento, frente ao temor gerado pela pandemia, eventuais dúvidas quanto aos sintomas associados a SCA e infecção por SARS-CoV-2 e problemas logísticos de atendimento gerados pelo colapso do sistema de saúde. No nosso serviço, por exemplo, 21 pacientes foram atendidos na emergência no protocolo gerenciado de dor torácica entre 20/03/2020 e 08/04/2020, compatível com uma redução relativa de 74% em relação ao mesmo período de 2019 e de 72% em relação ao mesmo período de 2018.

Uma série de casos oriunda de um único centro de atendimento de infarto agudo em Hong Kong demonstrou significativo atraso no atendimento destes pacientes em comparação com uma série histórica do ano anterior, com o aumento da mediana de tempo de todos os indicadores de qualidade assistencial analisados e em especial no tempo de início dos sintomas ao primeiro contato médico (318 minutos, IIQ 75 – 458 vs. 82,5 minutos, IIQ 32,5 – 195).^8^

### Suspensão do uso de ieca/bloqueador da angiotensina e risco de eventos

O paciente em questão havia suspenso por conta própria o uso do BRA. Apesar de não podermos definir um nexo causal entre a suspensão e a ocorrência do IAM, sabe-se que a descontinuação de medicações anti-hipertensivas pode contribuir para a maior ocorrência SCA .^9^

A enzima conversora de angiotensina do tipo 2 (ECA-2) parece estar imbricada no mecanismo de internalização do SARS-CoV-2 a nível tecidual. Esta informação gerou especulações de que usuários de inibidores da enzima conversora de angiotensina (IECA) ou de BRA pudessem ter maior chance de infectar-se devido a upregulation da ECA-2. Não existem dados clínicos publicados que comprovem esta relação fora a observação mecanicista, a não ser o racional teórico.^10^ Modelos experimentais em animais mostram efeitos inconsistentes de IECA e BRA sobre níveis de ECA-2 ou sua atividade tissular.^11^ Ademais, estudos transversais nos campos de insuficiência cardíaca, fibrilação atrial, estenose aórtica e doença coronariana^12^ resultaram em atividade plasmática de ECA-2 semelhante, independente do uso ou não de IECA e BRA. Além disso, o nível plasmático de ECA-2 pode não ser marcador confiável da forma ligada à membrana e faltam evidências de que modificação dos níveis de ECA-2 ou atividade nos tecidos favoreçam a penetração do SARS-CoV-2.

Neste cenário, as principais sociedades de cardiologia no mundo realizaram informativos e foram unânimes em orientar manutenção do uso dessas medicações, pois o risco de elevação rebote da pressão arterial ou de descompensação de quadros de insuficiência cardíaca poderiam acarretar potencial danoso maior.13 Vale ressaltar que alguns estudos preliminares até sugerem que estas medicações possam ter efeito protetor reduzindo a inflamação pulmonar.^14^

## Conclusão

No momento em que todos estão preocupados com os riscos potenciais da pandemia de COVID-19 precisamos estar atentos e alertar a população para que não deixe de valorizar sintomas sugestivos de eventos cardiovasculares e dos riscos relacionados à procura tardia de um atendimento de emergência. Os danos diretos do COVID-19 estão no topo da lista de discussões na mídia e nas revistas científicas, mas os potenciais danos cardiovasculares colaterais relacionados ao atendimento tardio de um paciente com evento vascular agudo não devem ser minimizados.

## References

[1] 1. Ibanez B, James S, Agewall S, Antunes M, Ducci CB, Alida HB, et al. 2017 ESC Guidelines for the management of acute myocardial infarction in patients presenting with ST-segment elevation. Eur Heart J. 2018;39(2):119-77. 10.1093/eurheartj/ehx393 28886621

[2] 2. Avezum Junior Á, Feldman A, Carvalho ACDC, Sousa ACC, Mansur AP, Bozza AEZ, et al. V Diretriz da Sociedade Brasileira de Cardiologia sobre Tratamento do Infarto Agudo do Miocárdio com Supradesnível do Segmento ST. Arq Bras Cardiol. 2015;105(2):1-105. 10.5935/abc.20150107 26375058

[3] 3. Driggin E, Madhavan M V, Bikdeli B, Chuich T, Laracy J, Zoccai GB, t al. et al. Cardiovascular Considerations for Patients, Health Care Workers, and Health Systems During the Coronavirus Disease 2019 (COVID-19) Pandemic. J Am Coll Cardiol. 2020;2019. 10.1016/j.jacc.2020.03.031 PMC7198856 32201335

[4] 4. Zeng J, Huang J, Pan L. How to balance acute myocardial infarction and COVID-19: the protocols from Sichuan Provincial People’s Hospital. Intensive Care Med. 2020;75(18):2352-371. 10.1007/s00134-020-05993-9 PMC7079823 32162032

[5] 5. Nguyen JL, Yang W, Ito K, Matte TD, Shaman J, Kinney PL. Seasonal influenza infections and cardiovascular disease mortality. JAMA Cardiol. 2016;1(3):274-81. 10.1001/jamacardio.2016.0433 PMC5158013 27438105

[6] 6. Bonow RO, Fonarow GC, O’Gara PT, Yancy CW. Association of Coronavirus Disease 2019 (COVID-19) With Myocardial Injury and Mortality. JAMA Cardiol. 2020;323(11):1061-9. 10.1001/jamacardio.2020.1105 32219362

[7] 7. Rodríguez-leor O, López-palop R, Serrador A, Martin-Moreiras J, Rumoroso JR, Perez de Prado A. Impacto de la pandemia de COVID-19 sobre la actividad asistencial en cardiología intervencionista en España.REC Interv Cardiol.2020;82-9.

[8] 8. Tam C-CF, Cheung K-S, Lam S,wang A, Yung A, Sza M, et al. Impact of Coronavirus Disease 2019 (COVID-19) Outbreak on ST-Segment–Elevation Myocardial Infarction Care in Hong Kong, China. Circ Cardiovasc Qual Outcomes. 2020;13(4):e006631,2020 04. 10.1161/CIRCOUTCOMES.120.006631 PMC7147280 32182131

[9] 9. Alharbi FF, Souverein PC, De Groot MC, Maitland-Van Der Zee AH, De Boer A, Klungel OH. Risk of acute myocardial infarction after discontinuation of antihypertensive agents: A case-control study. J Hum Hypertens. 2017;31(8):537-44. 10.1038/jhh.2017.1 28332511

[10] 10. Fang L, Karakiulakis G, Roth M. Are patients with hypertension and diabetes mellitus at increased risk for COVID-19 infection? Lancet Respir Med. 2020;8(4):e21. 10.1016/S2213-2600(20)30116-8 PMC7118626 32171062

[11] 11. Ferrario CM, Jessup J, Chappell MC, Averill DB, Brosniban AKB, Tallant A, et al. Effect of angiotensin-converting enzyme inhibition and angiotensin II receptor blockers on cardiac angiotensin-converting enzyme 2. Circulation. 2005;111(20):2605-10. 10.1161/CIRCULATIONAHA.104.510461 15897343

[12] 12. Ramchand J, Patel SK, Srivastava PM, Farouque O, Burrell LM. Elevated plasma angiotensin converting enzyme 2 activity is an independent predictor of major adverse cardiac events in patients with obstructive coronary artery disease. PLoS One. 2018;13(6):1-11. 10.1371/journal.pone.0198144 PMC5999069 29897923

[13] 13. Bavishi C, Maddox TM, Messerli FH. Coronavirus Disease 2019 (COVID-19) Infection and Renin Angiotensin System Blockers. JAMA Cardiol. 2020;19(8):1965-74. 10.1001/jamacardio.2020.1282 32242890

[14] 14. Kuba K, Imai Y, Rao S, Gao H, Guo F, Guan B, et al. A crucial role of angiotensin converting enzyme 2 (ACE2) in SARS coronavirus-induced lung injury. Nat Med. 2005;11(8):875-9. 10.1038/nm1267 PMC7095783 16007097

