# Supplementary material for: COVID-19 e Eventos Coronários Agudos – Danos Colaterais. Um Relato de Caso
Source: Arq Bras Cardiol. 2020 Jun 29;114(6):1072–5. [Article in Portuguese] doi: 10.36660/abc.20200329 (PMC8416134; doi:10.36660/abc.20200329)
Supplement: Translation

# Translation: COVID-19 and Acute Coronary Events – Collateral Damage. A Case Report.

**Authors:** Luiz Eduardo Fonteles Ritt, Mateus S. Viana, Gustavo Freitas Feitosa, Adriano Martins de Oliveira, Fabio Solano Souza, Eduardo Sahade Darzé

**Affiliations:** 1 Brazil Hospital Cardio Pulmonar, Salvador, BA - Brazil; 2 Brazil Escola Bahiana de Medicina e Saúde Pública, Salvador, BA - Brazil

**Keywords:** ST Myocardial Infarction, Coronavirus, Pandemics, Panic, Fear, Cineangiography, Echocardiography/methods, Risk Factors

*“On account of your fear, Sancho, you do not see or hear things correctly — said Don Quixote —, because one of the effects of fear is that it disturbs the senses and makes things seem not what they are.”*

Miguel de Cervantes, Don Quixote

A 49-year-old male patient, with dyslipidemia, 8-year-history of hypertension, and family history of coronary artery disease (His father had had an infarction at 60 years of age), had been using olmesartan 40 mg and rosuvastatin 10 mg daily until 10 days before being admitted to the hospital, having suspended use of olmesartan due to concern that the medication would facilitate SARS-CoV-2 infection.

On the morning of April 2, 2020, the patient had intense retrosternal chest discomfort and feeling of dyspnea. These symptoms were triggered by the slightest effort; they ceased while resting and recurred with decreasing intensity throughout the day. Concerned with the possibility of SARS-CoV-2 infection, he self-isolated, monitored his temperature, and self-administered paracetamol. He did not record a fever. The following day, chest pain recurred, radiating to his shoulders, in association with sweating and dyspnea. Due to the sweating, he became even more worried about the possibility of SARS-CoV-2, and he called an infectologist who instructed him to seek emergency medical care if the symptoms persisted or recurred. Throughout the day, the patient remained isolated and self-monitored his temperature. He reported that “only the possibility of coronavirus went through his mind.”

On the morning of April 4, when the pain worsened, and the sweating was more intense, the patient decided to seek emergency medical care. The case was screened as possible acute coronary syndrome (ACS), but the patient refused to undergo tests, because he did not wish to remain in the sector where there were other patients, and he left against medical advice. On his way home, symptoms intensified, namely more profuse sweating and dyspnea; the patient changed course and came to our hospital, where he presented with sinus tachycardia (HR 108 bpm), SBP 176 mmHg, O2 saturation 98%, and temperature 36.4ºC. Electrocardiogram revealed ST segment elevation in V5, V6, D1, and AVL ( Figure 1 ), indicating acute myocardial infarction with ST elevation (STEMI). The patient underwent coronary cineangiography and primary angioplasty in the middle third of the anterior descending artery, with a door-to-balloon time of 57 minutes ( Figure 2 ). Echocardiogram showed mild systolic dysfunction, due to akinesia of the entire apical region and the middle segment of the anterior wall; ejection fraction was 45%, using the Simpson Method. Peak high sensitivity troponin I was 21,424 ng/L. The patient progressed without complications and was discharged after 3 days of hospitalization. Figure 3 shows the timeline of events up to diagnosis of acute STEMI.

**Figure 1:** – *Electrocardiogram upon admission.*

**Figure 2:** – *Coronary cineangiography representing: a) occluded anterior descending coronary artery and b) after primary angioplasty.*

**Figure 3:** – *Timeline from onset of symptoms to diagnosis of myocardial infarction. ACS: acute coronary syndrome; ADA: anterior descending artery; ARB: angiotensin receptor blocker; CPH: Cardiopulmonary Hospital; ECG: electrocardiogram; ER: emergency room; SD: suspected diagnosis; STEMI: myocardial infarction with ST elevation.*

## Discussion

Considering the SARS-CoV-2 pandemic, quarantine periods have been declared in several cities in Brazil and worldwide, and people have been instructed to maintain social distancing in order to contain the rapid spread of the virus. Taken to the extreme, fear of becoming infected may result in typical symptoms of ACS being neglected or erroneously attributed to other less probable causes, delaying treatment and imposing avoidable risks to patients’ lives. We report a typical case of ACS in a patient with risk factors for atherosclerotic disease, who, driven by panic related to COVID-19, was unable to recognize the nature of his symptoms, thus delaying his trip to the emergency room until the moment that chest pain became unbearable. Furthermore, also due to concerns related to SARS-CoV-2 infection, the patient suspended use of angiotensin receptor blocker (ARB). In spite of a door-to-balloon time of 57 minutes, as a result of prolonged ischemia time, the patient developed left ventricular systolic dysfunction, albeit asymptomatic.

### Delayed recognition and medical care in acute myocardial infarction

Acute myocardial infarction (AMI) is the most lethal medical emergency worldwide, with an incidence of 43 – 144 per 100,000 people/year and a hospital mortality of 4% – 12%.^1^ Primary angioplasty, especially when instituted within the first 12 hours after onset of symptoms, is considered the gold standard treatment.^1 , 2^ Door-to-balloon time is an indicator of treatment quality in the context of AMI. It is equally important to minimize the time between onset of symptoms and arrival at a hospital. While time of attendance upon arrival at the hospital may be optimized by internal movement and protocol, time to arrival at the hospital depends almost exclusively on patients’ perception and evaluation of symptoms.

The SARS-CoV-2 pandemic has introduced other perspectives to this pathological approach, considering the potential risk of contamination in a hemodynamic environment, with procedures that may require more invasiveness, with inadequate environment for controlling the spread of the virus and guaranteeing the safety of healthcare professionals.^3^ A recent publication from the epicenter of the pandemic weighs the possibility of thrombolytic therapy for confirmed cases with respiratory symptoms of the disease.^4^

The reported case illustrates another scenario within the SARS-CoV-2 pandemic, which is as concerning as the pandemic itself. Previously published studies during other viral epidemics have suggested an increase in the occurrence of myocardial infarction, with a greater propensity for inflammation and plaque instability,^5^ and this also appears to be the rationale for SARS-CoV-2 infection.^6^ Nonetheless, reports in different world centers point to a reduction in the frequency of hospital admission due to infarction, with an observational study indicating a 40% decrease in attendance for STEMI, with a slight increase in the rate of thrombolysis.^7^ This paradoxical decline may be associated with a reduction in the number of patients seeking emergency care units, faced with fear generated by the pandemic, eventual doubts regarding symptoms associated with ACS and SARS-CoV-2 infection, and logistical issues related to healthcare caused by the collapse of the healthcare system. In our service, for instance, 21 patients were attended in the emergency room following the protocol for chest pain between March 20 and April 8, 2020; this is compatible with a 74% relative reduction with respect to the same period in 2019 and a 72% relative reduction with respect to the same period in 2018.

A case series from a single center for attending AMI in Hong Kong demonstrated a significant delay in providing care to these patients in comparison with a historical series from the previous year, with an increase in median time for all indicators of quality of care analyzed, especially time from onset of symptoms to first medical contact (318 minutes, IIQ 75 – 458 *vs.* 82.5 minutes, IIQ 32.5 – 195).^8^

### Suspension of angiotensin converting enzyme inhibitors/angiotensin receptor blockers and risk of events

The patient in question had suspended use of ARB of his own accord. Although we cannot define a causal nexus between this suspension and the occurrence of AMI, it is known that discontinuation of anti-hypertensive medications may contribute to greater occurrence of ACS.^9^ The type 2 angiotensin-converting enzyme (ACE-2) appears to be involved in the internalization mechanism of SARS-CoV-2 on the tissue level. This information has led to speculation that users of angiotensin-converting enzyme inhibitors (ACEI) or ARB may have a greater likelihood of becoming infected due to ACE-2 upregulation. There are no published clinical data to prove this relationship apart from mechanistic observation, except the theoretical rationale.^10^ Experimental models in animals have shown inconsistent effects of ACEI and ARB on levels of ACE-2 or its tissue activity.^11^ Furthermore, cross-sectional studies in the fields of heart failure, atrial fibrillation, aortic stenosis, and coronary disease^12^ resulted in similar ACE-2 plasma activity, regardless of whether ACEI and ARB were used or not. In addition to this, plasma levels of ACE-2 may not be reliable markers of the membrane-bound form, and there is a lack of evidence that modification of ACE-2 levels or tissue activity favor the penetration of SARS-CoV-2.

In this scenario, the world’s leading cardiology societies have published informational updates, unanimously advising people to the maintain the use of these medications, given that the risk of rebound high blood pressure or decompensation of heart failure could lead to greater potential harm.^13^ It is worth underscoring that some preliminary studies have even suggested that these medications may have a protective effect, reducing pulmonary inflammation.^14^

## Conclusion

At this time, when everyone is concerned with the potential risks of the COVID-19 pandemic, we need to be aware and alert the population not to underestimate symptoms that are suggestive of cardiovascular events or risks related to delays in seeking emergency medical care. The direct harm of COVID-19 is at the center of media discussions and scientific publications, but the potential cardiovascular collateral damage related to delayed medical care in patients with acute vascular events should not go neglected.
